# The influence of blood donation before pregnancy on neonatal birth weight

**DOI:** 10.1371/journal.pone.0269367

**Published:** 2022-06-24

**Authors:** Genjie Lu, Zhe Zhu, Yangfang Lu, Jun Shen, Qilin Yu, Li Gao, Wei Chen

**Affiliations:** 1 Department of Blood Transfusion, Ningbo Medical Center Lihuili Hospital, Ningbo University, Ningbo, China; 2 Department of Blood Transfusion, HwaMei Hospital, University of Chinese Academy of Sciences, Ningbo, China; 3 Department of Radiotherapy, Ningbo Medical Center Lihuili Hospital, Ningbo University, Ningbo, China; 4 Department of Obstetrics, Ningbo Medical Center Lihuili Hospital, Ningbo University, Ningbo, China; University of Cambridge, UNITED KINGDOM

## Abstract

**Objective:**

To evaluate the effect of blood donation before pregnancy on neonatal birth weight.

**Methods:**

A total of 14996 women with singleton pregnancies at full-term in Ningbo Medical Center Lihuili Hospital and Ningbo Women’s and Children’s Hospital from November 2019 to November 2020 were enrolled in this study. Detailed records of whole blood donation before pregnancy were obtained through Alipay software. The records were classified into three groups: nondonors, low-frequency donors and high-frequency donors according to the total numbers of blood donations in the 3-year period before pregnancy. The demographics and clinical information of the enrolled participants and their fetuses were collected from electronic medical records (EMRs). The effect of blood donations in the 3-year period before pregnancy on neonatal birth weight was analyzed.

**Results:**

There was no significant difference in neonatal birth weight among the three groups (*P* = 0.373). In line with this, there was no association between the number of blood donations in the 3-year period before pregnancy and neonatal birth weight (β = 14.5; 95% confidence interval [CI]: -3.9, 31.4; *P* = 0.094) in the bootstrapped multivariate linear regression analysis models, adjusted for maternal age, number of pregnancies, number of deliveries, gestational age, mode of delivery, years of education and blood type in pregnant women. Compared to the nondonors, the risk of fetal macrosomia was higher in both low-frequency donors and all donors (OR: 1.539, 95% CI: 1.058, 2.134, P = 0.016; OR: 1.454, 95% CI: 1.033, 1.952, P = 0.021, respectively), in the bootstrapped binary logistic regression analysis models after adjusting for the variables mentioned above.

**Conclusion:**

Our study showed that maternal blood donation in the 3-year period before pregnancy may not lead to a reduction in neonatal birth weight, but may be associated with the incidence of fetal macrosomia.

## Introduction

Iron is essential for some physiological processes [1]. Iron deficiency will cause anemia [2, 3] and contribute to the poor prognosis of heart failure, etc. [4]. Diet and physiological factors play a key role in iron status [5–7]; however, it may also be affected by blood donation [8–12]. Rigas et al. [13] found that the number of blood donations in a 3-year period was one of the strongest predictor of iron deficiency. Iron deficiency caused by frequent blood donation is extremely common in female blood donors, and it was observed that 39% of premenopausal women who donate blood frequently have iron deficiency [13].

Intervals between blood donations range from 8 weeks to 6 months in different countries, which may cause varied degrees of iron recovery. In our country, the interval between whole blood donations must be more than 6 months. Although iron is not detected directly, hemoglobin must be measured before blood donation. If signs of anemia are discovered, blood donation must be postponed until hemoglobin returns to normal.

It has been found that iron deficiency during pregnancy is associated with an increased risk of small for gestation infants [14] and low birthweight [15]. Iron deficiency can be a risk factor for intrauterine growth retardation [14]. In addition, an animal study demonstrated that low iron stores in pregnancy had adverse effects on brain development in infants [16]. Therefore, iron deficiency in pregnancy caused by blood donation may have adverse effects on fetal health and development.

To date, few studies have directly focused on the effect of blood donation before pregnancy on neonatal birth weight, with slightly different results reported [17–19]. A Danish study [17] observed that high-intensity blood donation before pregnancy was inversely associated with fetal birth weight, which was not reported in Canadian studies [18, 19]. A previous study in Canada [18] observed a reduced risk of low birthweight in donors, which was not discovered in the Danish study [17] or another Canadian study [19]. Therefore, further studies are warranted. To better understand the influence of maternal blood donations on neonatal birth weight in China, a multicenter, prospective cohort study was performed in Ningbo, Zhejiang Province.

## Materials and methods

### Date collection and study design

This was a multicenter, prospective cohort study performed at Ningbo Medical Center Lihuili Hospital and Ningbo Women’s and Children’s Hospital from November 2019 to November 2020.

In this study, women with singleton pregnancies at full-term (gestational ≥ 37 weeks) were eligible for inclusion. After 4 women were excluded because they were less than 18 years old, 14996 pregnant women were enrolled in the study. The detailed records of whole blood donation of women in Zhejiang Province before pregnancy were obtained through Alipay software v10.1 (Alipay (China) Network Technology Co., Ltd., China). The demographics and clinical information, including maternal age, number of pregnancies, number of deliveries, gestational age, mode of delivery (vaginal or caesarean delivery), years of education and blood type in pregnant women, and fetal sex and birth weight, were obtained by electronic medical records (EMR, KINGT software v5.0) (Ningbo Jintang Software Co., Ltd, China).

A previous study found that blood type is associated with ferritin levels in blood donors [7]; therefore, we included blood type as a confounding variable. Additionally, whether women smoked during pregnancy may influence the results [17], but it was excluded because none of the participants smoked during pregnancy in our research study. In addition, as neonatal birth weight is closely associated with gestational age [17], pregnant women were only selected at least 37 weeks of gestational age, that is, at term.

In light of the fact that blood donors were allowed to donate whole blood up to two times per year in China, women were stratified into three groups according to the total number of blood donations in the 3-year period before pregnancy: nondonors who had no donation activity, low-frequency donors who provided 1–2 donations, and high-frequency donors who provided at least 3 donations.

According to fetal birth weight, newborns were divided into 3 groups: low birthweight (<2500 g), normal birthweight (2500 g– 3999 g) and macrosomia (≥4000 g) [19, 20].

During the recruitment process, an invitation was sent to potential qualified pregnant women interested in participating in the study at the doctor’s office, where the doctor obtained verbally informed consent, and mobile recording was conducted simultaneously. The notification and consent included instructions to conduct the research, risk determination, maternal blood donation data procurement, clinical information, fetal sex and birth weight, confidentiality of information, etc. This study was approved by the ethics committee of Ningbo Medical Center Lihuili Hospital (Approval no. KY2019PJ020) and was conducted in a designated clinical environment.

### Statistical analysis

Categorical variables were reported as number (%), and significance was tested by the chi-square test. Abnormally distributed variables were expressed as medians and interquartile ranges (IQRs), and significance was detected by the Kruskal–Wallis H test. A bootstrapped multivariate linear regression analysis of fetal birth weight as an outcome was performed by taking the number of donations before pregnancy and all potential confounders (maternal age, number of pregnancies, number of deliveries, gestational age, mode of delivery, years of education and blood type of pregnant women) as independent variables. Bootstrapped binary logistic regression analysis was performed to assess the association of the frequency of donations before pregnancy and fetal macrosomia while controlling all potential confounders as mentioned above. A *P* value less than 0.05 was considered statistically significant. All data were analyzed by SPSS statistical software, version 19.0 (IBM, Armonk, NY, USA).

## Results

A total of 14996 pregnant women who delivered from November 2019 to November 2020 were included in this study. Detailed records of whole blood donation for the patients in Zhejiang Province before pregnancy were obtained by Alipay software (Table 1). The blood donation rates in the 3-year period before pregnancy and in the entire time before pregnancy were 4.6% and 14.5%, respectively (Table 1). The rates of non-donors, low-frequency donors, and high-frequency donors were 95.4%, 4.3%, and 0.3% in the 3-year period before pregnancy, respectively (Table 2).

**Table 1:** Frequency distribution of the number of whole blood donations before pregnancy in Zhejiang Province, China.

Number of donations	Donation in the 3-year period before pregnancy[n(%)]	Donation in the entire time before pregnancy[n(%)]
**0**	14313(95.4)	12819(85.5)
**1**	511(3.4)	1547(10.3)
**2**	128(0.9)	380(2.5)
**3**	26(0.2)	129(0.9)
**4**	18(0.1)	58(0.4)
**5**	0(0.0)	35(0.2)
**6**	0(0.0)	15(0.1)
**7**	0(0.0)	4(0.0)
**8**	0(0.0)	7(0.0)
**10**	0(0.0)	2(0.0)
**Total**	14996(100.0)	

**Table 2:** Demographic and clinical characteristics according to blood donation in the 3-year period before pregnancy.

	Total	Nondonors	Low frequency donors	High frequency donors	*P* value †
Characteristics	Median (IQR) or n (%)				
Number (%)	14996 (100.0)	14313 (95.4)	639 (4.3)	44 (0.3)	
Maternal age (years)	29.0 (26.0–32.0)	29.0 (27.0–32.0)	28.0 (26.0–31.0)	29.5 (28.0–34.0)	<0.001
Number of pregnancies	2 (1–3)	2 (1–3)	2 (1–3)	2 (1–2)	<0.001
Number of deliveries	1 (1–2)	1 (1–2)	1 (1–2)	1 (1–2)	<0.001
Gestational age (weeks)	39 (38–40)	39 (38–40)	39 (38–40)	39 (38–39)	0.900
Natural birth	9538 (63.6)	9102 (63.6)	417 (65.3)	19 (43.2)	0.001
Years of education (years)	15(12–16)	15(12–16)	16(15–16)	16(16–16)	<0.001
O blood type	4833 (32.2)	4614 (32.2)	205 (32.1)	14 (31.8)	0.992
Fetal birth weight(g)	3350 (3100–3600)	3350 (3100–3600)	3350 (3100–3650)	3350 (3150–3800)	0.373
Fetal sex (male)	7837 (52.3)	7474 (52.2)	344 (53.8)	19 (43.2)	0.169
Low birthweight	141 (0.9)	137 (1.0)	3 (0.5)	1 (2.3)	0.088
Fetal macrosomia	892 (5.9)	839 (5.9)	51 (8.0)	2 (4.5)	0.055

Nondonors were defined as having no donation activity in the 3-year period, low-frequency donors were defined as having 1–2 donations in the 3-year period before pregnancy, and high-frequency donors were defined as having ≥3 donations in the 3-year period before pregnancy.

Data are presented as n (%) for categorical variables and median (IQR) for continuous variables.

† Kruskal–Wallis H test for continuous variables, and Chi-square test for categorical variables.

The maternal median age was 29.0 years (Table 2). There was a statistically significant difference in maternal age (*P*<0.001), number of pregnancies (*P*<0.001), number of deliveries (*P*<0.001), years of education (*P*<0.001), and natural birth (*P* = 0.001) among the three groups (nondonors, low-frequency donors, and high-frequency donors) (Table 2). However, no significant difference was observed in the gestational age (*P* = 0.900) and blood type (*P* = 0.992) of pregnant women or fetal sex (*P* = 0.169) among the three groups (Table 2).

In the univariate analysis, it was observed that the median fetal birthweight was 3350 g, and there was no significant difference between the three groups (*P* = 0.373) (Table 2). In line with this, there was no association between the number of donations in the 3-year period before pregnancy and neonatal birth weight in the bootstrapped multivariate linear regression analysis models, adjusted for maternal age, number of pregnancies, number of deliveries, gestational age, mode of delivery, years of education and blood type of pregnant women (β = 14.5; 95% confidence interval [CI]: -3.9, 31.4; *P* = 0.094) (Table 3).

**Table 3:** Linear regression analysis with fetal birth weight (g) as an outcome by the variables including the donation number in the 3-year period (β-values and 95% confidence intervals that were estimated with bootstrapping).

Variables	β	95% CI	*P* value
Constant	-2343.9	-2584.5, -2106.0	0.001
Maternal age (one extra year)	3.140	1.4, 4.7	0.001
Number of pregnancies (one extra pregnancy)	9.3	3.2, 16.0	0.005
Number of deliveries (one extra delivery)	53.7	37.3, 67.6	0.001
Gestational age (one extra week)	142.0	136.3, 147.8	0.001
Natural birth	-87.4	-100.2, -74.5	0.001
Years of education (one extra year)	3.1	0.9, 5.4	0.010
O blood type	2.0	-9.5, 14.9	0.725
The number of blood donation in the 3-year period before pregnancy (one extra donation)	14.5	-3.9, 31.4	0.094

Multivariable linear regression was adjusted for maternal age, number of pregnancies, number of deliveries, gestational age, mode of delivery, years of education, blood type and number of blood donation in the 3-year period before pregnancy.

Bootstrap results were based on 1000 bootstrap samples.

CI = confidence interval.

In the univariate analysis, the occurrence of fetal macrosomia was not significantly different between the three groups (*P* = 0.055) (Table 2). In contrast, in the bootstrapped binary logistic regression analysis models, both low-frequency donors and all donors had a higher incidence of fetal macrosomia than nondonors (OR: 1.539, 95% CI: 1.058, 2.134, *P* = 0.016; OR: 1.454, 95% CI: 1.033, 1.952, *P* = 0.021, respectively) after adjusting for maternal age, number of pregnancies, number of deliveries, gestational age (≥39 weeks), natural birth, years of education (≥15 years) and O blood type of pregnant women (Table 4). However, the case counts for fetal macrosomia (Table 2) showed that there were insufficient data for high-frequency donors to fit the binary logistic regression analysis models.

**Table 4:** Binary logistic regression of variables associated with fetal macrosomia (odds ratios and 95% confidence intervals that were estimated with bootstrapping).

	Model 1			Model 2		
Variables	OR	95% CI	*P*	OR	95% CI	*P*
Maternal age	1.021	0.998, 1.042	0.061	1.021	0.998, 1.043	0.062
Number of pregnancies	0.996	0.912, 1.085	0.916	1.004	0.908, 1.088	0.933
Number of deliveries	1.294	1.063, 1.562	0.009	1.281	1.065, 1.548	0.016
Gestational age(≥39 weeks)	1.785E+08	1.625E+08, 1.949E+08	0.001	1.783E+08	1.620E+08, 1.947E+08	0.001
Natural birth	0.484	0.414, 0.568	0.001	0.486	0.417, 0.569	0.001
Years of education (≥15 years)	1.041	0.860, 1.274	0.680	1.041	0.850, 1.290	0.698
O blood type	1.017	0.860, 1.197	0.854	1.011	0.856, 1.203	0.894
Blood donation						
Nondonors	-	-	-	-	-	-
Low-frequency donors	1.539	1.058, 2.134	0.016			
Donors				1.454	1.033, 1.952	0.021
Constant	2.806E-10	1.579E-10, 4.977E-10	0.001	2.778E-10	1.562E-10, 4.908E-10	0.001

Nondonors were defined as having no donation activity in the 3-year period before pregnancy; low-frequency donors were defined as having 1–2 donations in the 3-year period before pregnancy; high-frequency donors were defined as having ≥3 donations in the 3-year period before pregnancy; donors were defined as having ≥1 donation in the 3-year period before pregnancy, including low-frequency and high-frequency donors.

Model 1 and Model 2 for fetal macrosomia adjusted for maternal age, number of pregnancies, number of deliveries, gestational age (≥39 weeks), natural birth, years of education (≥15 years), O blood type and blood donation (Model 1: low-frequency donors compared to nondonors; Model 2: all donors compared to nondonors).

Bootstrap results were based on 1000 bootstrap samples.

OR = odd ratio; CI = confidence interval.

In the univariate analysis, the risk of low birthweight was not significantly different between the three groups (*P* = 0.088) (Table 2). However, the case counts for low birthweight (Table 2) showed that there were insufficient data for low-frequency donors, high-frequency donors or all donors to fit the binary logistic regression analysis models, which could determine their risk of low birthweight compared to nondonors.

## Discussion

In this multicenter, prospective cohort study, we found that there was no association between blood donation in the 3-year period before pregnancy and neonatal birth weight. On the other hand, the incidence of fetal macrosomia in both low-frequency donors and all donors was higher than that in nondonors, which was an interesting discovery.

Because blood donation may affect iron stores [13] and cause iron deficiency during pregnancy which may increase the risk of small for gestational age infant [14], some studies have directly focused on the effect of blood donation before pregnancy on neonatal birth weight. For example, a Danish study [17] observed that high- intensity blood donation before pregnancy was inversely associated with neonatal birth weight, while it was not found in our study or Canadian studies [18, 19]. In addition, a previous study in Canada observed that donors had a reduced risk of low birthweight [18], which was not found in the Danish study [17] or another Canadian study [19]. Unfortunately, because the number of low birthweights in low-frequency donors, high-frequency donors and all donors were too small in our study, we could not further analyze their risk of low birthweight compared with nondonors.

In addition, the rates of fetal macrosomia were reported to be higher in donors than in nondonors (13.7% vs. 10.8%) in a previous study [18], although a statistical analysis between them was not performed. In line with this, it was interesting that we found an increased risk of fetal macrosomia in low-frequency donors and all donors compared with non-donors. However, the numbers of fetal macrosomia in low-frequency and high-frequency donors were very small, especially in high-frequency donors, which may cause results bias. Therefore, we combined high-frequency and low-frequency donors together for a subset analysis of donor groups to improve the accuracy of the results. However, additional studies are needed for further validation.

In our opinion, the following reasons may account for the differences. First, blood donation policies in different countries are diverse [21–24]. For example, donors are allowed to have up to six donation activities per year in North America and Canada [19], while it is only allowed twice, at most, in China. The recovery of iron-related indicators in blood donors takes a long time, even more than 6 months [25]; thus, the recovery of iron status due to blood donation policies may play a key role. Second, the willingness to donate blood is different. The rate of donation in the 3-year period before pregnancy in our study was only 4.6%, which was lower than 58.7% in Canada [19] (in the 2-year period before pregnancy) and 8.7% in Denmark [17]. Third, the confounding variables in each study were different, which may cause results bias.

There are several strengths in our research. First, blood donation records were obtained from maternal Alipay software. On January 16, 2018, the Zhejiang Blood Center cooperated with Alipay software to launch the first electronic blood donation card on the Alipay platform in China. Individuals can query the exact blood donation data and blood destinations in Zhejiang Province by Alipay. Therefore, we took advantage of Alipay to research the frequency of blood donations before pregnancy and neonatal birth weight directly on a large local scale. To the best of our knowledge, this is the first study using the patient’s Alipay to obtain blood donation history. In addition, and to the best of our knowledge, we conducted a direct study on blood donation before pregnancy and neonatal birth weight, which is the first relevant study in China.

However, there are some limitations in our study. First, due to the backwardness of the blood donation information system, the records of blood donation were only obtained from Zhejiang Province and not from the entire country in the study period. Fortunately, the history of donations in the entire country is readily available by Alipay, which is also the direction of our follow-up research. Second, demographics and clinical information, such as pre-pregnancy body mass index and detailed paternal information were not sufficiently obtained from EMR. Third, the association between the amount of donated blood and fetal birth weight was not analyzed. Fourth, the sample size in the study was relatively small, especially of low birthweight in low-frequency and high-frequency donors and fetal macrosomia in high-frequency donors, so it may not be representative of a larger population. Therefore, more multicenter studies with larger sample sizes are warranted.

## Conclusion

Our study showed that blood donation in the 3-year period before pregnancy may not lead to a reduction in neonatal birth weight, but may be associated with the incidence of fetal macrosomia. However, more research is needed to further verify this hypothesis.

## Supporting information

S1 File Logistic regression assumptions for Table 4. (PDF)
