# Supplementary material for: The influence of blood donation before pregnancy on neonatal birth weight
Source: PLoS One. 2022 Jun 24;17(6):e0269367. doi: 10.1371/journal.pone.0269367 (PMC9231744; doi:10.1371/journal.pone.0269367)
Supplement: S1 File — (PDF) [file pone.0269367.s001.pdf]

# Logistic regression assumptions for Table 4

## Linearity assumption

Here, we’ll check the linear relationship between continuous predictor variables and the logit of the outcome by Box-Tidwell method. This can be done by incorporating the interaction between the continuous independent variable and its natural logarithm into the regression equation.

1.

**Variables in the Equation**

A

| | | B | S.E. | Wald | df | Sig. | Exp(B) |
|---|---|---|---|---|---|---|---|
| Step 1[a] | Maternalage | 1.126 | .395 | 8.112 | 1 | .004 | 3.082 |
| | Numberofpregnancies | .122 | .226 | .291 | 1 | .589 | 1.130 |
| | Numberofdeliveries | .430 | .554 | .604 | 1 | .437 | 1.537 |
| | Gestationalage | 34.674 | 9.838 | 12.422 | 1 | .000 | 1.145E15 |
| | Naturalbirth(1) | -.769 | .074 | 107.545 | 1 | .000 | .463 |
| | Yearsofeducation | 1.841 | .478 | 14.808 | 1 | .000 | 6.300 |
| | Obloodtype(1) | -.129 | .077 | 2.791 | 1 | .095 | .879 |
| | LowfrequencyDonors(1) | .395 | .154 | 6.562 | 1 | .010 | 1.484 |
| | Maternalage by ln_Maternalage | -.250 | .090 | 7.802 | 1 | .005 | .779 |
| | Numberofpregnancies by ln_Numberofpregnancies | -.045 | .108 | .176 | 1 | .675 | .956 |
| | Numberofdeliveries by ln_Numberofdeliveries | -.134 | .352 | .145 | 1 | .703 | .874 |
| | Gestationalage by ln_Gestationalage | -7.280 | 2.107 | 11.936 | 1 | .001 | .001 |
| | Yearsofeducation by ln_Yearsofeducation | -.519 | .135 | 14.778 | 1 | .000 | .595 |
| | Constant | -329.723 | 82.533 | 15.961 | 1 | .000 | .000 |

a. Variable(s) entered on step 1: Maternalage, Numberofpregnancies, Numberofdeliveries, Gestationalage, Naturalbirth, Yearsofeducation, Obloodtype, LowfrequencyDonors, Maternalage * ln_Maternalage , Numberofpregnancies * ln_Numberofpregnancies , Numberofdeliveries * ln_Numberofdeliveries , Gestationalage * ln_Gestationalage , Yearsofeducation * ln_Yearsofeducation .

**Variables in the Equation**

B

| | | B | S.E. | Wald | df | Sig. | Exp(B) |
|---|---|---|---|---|---|---|---|
| Step 1[a] | Maternalage | 1.069 | .392 | 7.426 | 1 | .006 | 2.913 |
| | Numberofpregnancies | .113 | .225 | .250 | 1 | .617 | 1.119 |
| | Numberofdeliveries | .454 | .553 | .672 | 1 | .413 | 1.574 |
| | Gestationalage | 35.191 | 9.984 | 12.424 | 1 | .000 | 1.919E15 |
| | Naturalbirth(1) | -.768 | .074 | 107.404 | 1 | .000 | .464 |
| | Yearsofeducation | 1.842 | .478 | 14.863 | 1 | .000 | 6.309 |
| | Obloodtype(1) | -.134 | .077 | 3.013 | 1 | .083 | .875 |
| | Donors(1) | .350 | .151 | 5.348 | 1 | .021 | 1.419 |
| | Maternalage by ln_Maternalage | -.238 | .089 | 7.126 | 1 | .008 | .789 |
| | Numberofpregnancies by ln_Numberofpregnancies | -.038 | .107 | .123 | 1 | .726 | .963 |
| | Numberofdeliveries by ln_Numberofdeliveries | -.151 | .352 | .184 | 1 | .668 | .860 |
| | Gestationalage by ln_Gestationalage | -7.390 | 2.138 | 11.944 | 1 | .001 | .001 |
| | Yearsofeducation by ln_Yearsofeducation | -.519 | .135 | 14.822 | 1 | .000 | .595 |
| | Constant | -333.724 | 83.757 | 15.876 | 1 | .000 | .000 |

a. Variable(s) entered on step 1: Maternalage, Numberofpregnancies, Numberofdeliveries, Gestationalage, Naturalbirth, Yearsofeducation, Obloodtype, Donors, Maternalage * ln_Maternalage , Numberofpregnancies * ln_Numberofpregnancies , Numberofdeliveries * ln_Numberofdeliveries , Gestationalage * ln_Gestationalage , Yearsofeducation * ln_Yearsofeducation .

A: Model 1 in Table 4; B: Model 2 in Table 4.

In this study, a total of 14 items were included in the model analysis, containing five continuous independent variables including maternal age, number of pregnancies, number of deliveries, gestational age, years of education; three categorical independent variable including natural birth, O blood type, low-frequency donors /donors in the 3 year period before pregnancy; five interaction items including maternal age*ln_maternal age, number of pregnancies*ln_number of pregnancies, number of deliveries*ln_number of deliveries, gestational age*ln_gestational age, years of education*ln_ years of education and intercept term(constant).

Therefore, in this study, it was suggested that the significance level should be α = 00357 (i.e. 0.05 ÷ 14). According to the significance level, the *P* values of two interaction items including gestational age*ln_gestational age and years of education*ln_years of education in this study were less than 0.00357, three interaction items including maternal age*ln_maternal age , number of pregnancies*ln_number of pregnancies, number of deliveries*ln_number of deliveries were higher than 0.00357, so we must transform gestational age and years of education into classification variables.

2. Then, gestational age and years of education were transformed into classification

variables: gestational age (≥ 39 weeks or not), years of education (≥ 15 years or not) respectively.

C

**Variables in the Equation**

| | | B | S.E. | Wald | df | Sig. | Exp(B) |
|---|---|---|---|---|---|---|---|
| Step 1[a] | Maternalage | 1.097 | .392 | 7.851 | 1 | .005 | 2.996 |
| | Numberofpregnancies | .205 | .224 | .833 | 1 | .361 | 1.227 |
| | Numberofdeliveries | .199 | .554 | .129 | 1 | .720 | 1.220 |
| | Gestationalage≥39weeks(1) | 1.327 | .092 | 207.969 | 1 | .000 | 3.770 |
| | Naturalbirth(1) | -.727 | .073 | 98.865 | 1 | .000 | .483 |
| | Yearsofeducation≥15years(1) | .043 | .087 | .240 | 1 | .624 | 1.044 |
| | Obloodtype(1) | -.125 | .077 | 2.671 | 1 | .102 | .882 |
| | LowfrequencyDonors(1) | .366 | .153 | 5.720 | 1 | .017 | 1.441 |
| | Maternalage by In_Maternalage | -.246 | .089 | 7.639 | 1 | .006 | .782 |
| | Numberofpregnancies by In_Numberofpregnancies | -.087 | .107 | .661 | 1 | .416 | .917 |
| | Numberofdeliveries by In_Numberofdeliveries | -.022 | .353 | .004 | 1 | .950 | .978 |
| | Constant | -11.618 | 2.681 | 18.777 | 1 | .000 | .000 |

a. Variable(s) entered on step 1: Maternalage, Numberofpregnancies, Numberofdeliveries, Gestationalage≥39weeks, Naturalbirth, Yearsofeducation≥15years, Obloodtype, LowfrequencyDonors, Maternalage * In_Maternalage , Numberofpregnancies * In_Numberofpregnancies , Numberofdeliveries * In_Numberofdeliveries .

D

**Variables in the Equation**

| | | B | S.E. | Wald | df | Sig. | Exp(B) |
|---|---|---|---|---|---|---|---|
| Step 1[a] | Maternalage | 1.046 | .389 | 7.240 | 1 | .007 | 2.848 |
| | Numberofpregnancies | .189 | .223 | .720 | 1 | .396 | 1.208 |
| | Numberofdeliveries | .227 | .554 | .168 | 1 | .682 | 1.255 |
| | Gestationalage≥39weeks(1) | 1.329 | .092 | 209.078 | 1 | .000 | 3.778 |
| | Naturalbirth(1) | -.724 | .073 | 98.458 | 1 | .000 | .485 |
| | Yearsofeducation≥15years(1) | .045 | .087 | .268 | 1 | .605 | 1.046 |
| | Obloodtype(1) | -.129 | .077 | 2.849 | 1 | .091 | .879 |
| | Donors(1) | .312 | .150 | 4.320 | 1 | .038 | 1.366 |
| | Maternalage by In_Maternalage | -.234 | .088 | 7.035 | 1 | .008 | .791 |
| | Numberofpregnancies by In_Numberofpregnancies | -.077 | .106 | .522 | 1 | .470 | .926 |
| | Numberofdeliveries by In_Numberofdeliveries | -.042 | .353 | .014 | 1 | .905 | .959 |
| | Constant | -11.297 | 2.664 | 17.977 | 1 | .000 | .000 |

a. Variable(s) entered on step 1: Maternalage, Numberofpregnancies, Numberofdeliveries, Gestationalage≥39weeks, Naturalbirth, Yearsofeducation≥15years, Obloodtype, Donors, Maternalage * In_Maternalage , Numberofpregnancies * In_Numberofpregnancies , Numberofdeliveries * In_Numberofdeliveries .

C: Model 1 in Table 4; D: Model 2 in Table 4.

A total of 12 items were included in the model analysis, containing three continuous independent variables including maternal age, number of pregnancies, number of deliveries; five categorical independent variable including gestational age (≥ 39 weeks), natural birth, years of education (≥ 15 years), O blood type, low-frequency donors /donors in the 3 year period before pregnancy; three interaction items including maternal age*ln_maternal age , number of pregnancies*ln_number of pregnancies , number of deliveries*ln_number of deliveries and intercept term(constant).

Therefore, in this study, it was suggested that the significance level should be α = 00417 (i.e. 0.05 ÷ 12). According to the significance level, the *P* values of all interaction items in Table 4 were higher than 0.00417, so there was a linear relationship between the logit conversion values of all continuous independent variables and dependent variables.

**Multicollinearity**

Multicollinearity corresponds to a situation where the data contain highly correlated predictor variables.

**Coefficients[a]**

| Model | | Collinearity Statistics Tolerance | VIF |
|---|---|---|---|
| 1 | Maternal age | .680 | 1.470 |
| | Number of pregnancies | .482 | 2.075 |
| | Number of deliveries | .474 | 2.108 |
| E | Gestational age (≥39weeks) | .920 | 1.087 |
| | Natural birth | .908 | 1.101 |
| | Years of education (≥15years) | .812 | 1.232 |
| | O blood type | .999 | 1.001 |
| | Low-frequency Donors | .994 | 1.006 |

a. Dependent Variable: Macrosomia

**Coefficients[a]**

| Model | | Collinearity Statistics Tolerance | VIF |
|---|---|---|---|
| 1 | Maternal age | .680 | 1.471 |
| | Number of pregnancies | .482 | 2.074 |
| | Number of deliveries | .474 | 2.108 |
| F | Gestational age (≥39weeks) | .920 | 1.087 |
| | Natural birth | .908 | 1.101 |
| | Years of education (≥15years) | .811 | 1.233 |
| | O blood type | .999 | 1.001 |
| | Donors | .993 | 1.007 |

a. Dependent Variable: Macrosomia

E: Model 1 in Table 4; F: Model 2 in Table 4.

If the tolerance is less than 0.1 or the vif is greater than 10, then there is collinearity. In this study, the tolerances were much greater than 0.1, and the variance expansion factors were less than 10, so there were no multicollinearity.

**Influential values**

Influential values are extreme individual data points that can alter the quality of the logistic regression model.

**Casewise List[b]**

G-1

| Case | Selected Status[a] | Observed | Predicted | Predicted Group | Temporary Variable | |
|---|---|---|---|---|---|---|
| | | Macrosomia | | | Resid | ZResid |
| 202 | S | 1** | .038 | 0 | .962 | 5.052 |
| 337 | S | 1** | .019 | 0 | .981 | 7.225 |
| 345 | S | 1** | .041 | 0 | .959 | 4.813 |
| 359 | S | 1** | .042 | 0 | .958 | 4.791 |
| 454 | S | 1** | .029 | 0 | .971 | 5.738 |
| 624 | S | 1** | .041 | 0 | .959 | 4.813 |
| 795 | S | 1** | .032 | 0 | .968 | 5.498 |
| 917 | S | 1** | .020 | 0 | .980 | 6.913 |
| 934 | S | 1** | .024 | 0 | .976 | 6.352 |
| 1022 | S | 1** | .038 | 0 | .962 | 5.056 |
| 1070 | S | 1** | .040 | 0 | .960 | 4.898 |
| 1185 | S | 1** | .032 | 0 | .968 | 5.498 |
| 1412 | S | 1** | .040 | 0 | .960 | 4.898 |
| 1415 | S | 1** | .024 | 0 | .976 | 6.352 |
| 1497 | S | 1** | .029 | 0 | .971 | 5.738 |
| 1720 | S | 1** | [illegible] | 0 | .981 | 7.225 |
| 1799 | S | 1** | [illegible] | 0 | .981 | 7.106 |
| 1972 | S | 1** | [illegible] | 0 | .981 | 7.225 |
| 2155 | S | 1** | .020 | 0 | .980 | 6.913 |
| 2323 | S | 1** | .038 | 0 | .962 | 5.056 |
| 2375 | S | 1** | .020 | 0 | .980 | 6.913 |
| 2541 | S | 1** | .038 | 0 | .962 | 5.052 |
| 2697 | S | 1** | .019 | 0 | .981 | 7.106 |
| 2795 | S | 1** | .038 | 0 | .962 | 5.052 |
| 2824 | S | 1** | .040 | 0 | .960 | 4.898 |
| 2973 | S | 1** | .042 | 0 | .958 | 4.791 |
| 3111 | S | 1** | .038 | 0 | .962 | 5.056 |
| 3144 | S | 1** | .039 | 0 | .961 | 4.985 |
| 3157 | S | 1** | .033 | 0 | .967 | 5.431 |
| 3215 | S | 1** | .020 | 0 | .980 | 6.944 |
| 3261 | S | 1** | .033 | 0 | .967 | 5.431 |
| 3315 | S | 1** | .039 | 0 | .961 | 4.985 |
| 3494 | S | 1** | .037 | 0 | .963 | 5.095 |
| 3540 | S | 1** | .020 | 0 | .980 | 6.944 |
| 3585 | S | 1** | .020 | 0 | .980 | 6.944 |
| 3692 | S | 1** | .033 | 0 | .967 | 5.431 |
| 3812 | S | 1** | .037 | 0 | .963 | 5.095 |

G-2

| | | | | | | |
|---|---|---|---|---|---|---|
| 3812 | S | 1** | .037 | 0 | .963 | 5.095 |
| 3901 | S | 1** | .038 | 0 | .962 | 5.056 |
| 3921 | S | 1** | .037 | 0 | .963 | 5.095 |
| 4305 | S | 1** | .015 | 0 | .985 | 8.002 |
| 4358 | S | 1** | .015 | 0 | .985 | 8.002 |
| 4456 | S | 1** | .041 | 0 | .959 | 4.812 |
| 4528 | S | 1** | .021 | 0 | .979 | 6.778 |
| 4673 | S | 1** | .034 | 0 | .966 | 5.342 |
| 4690 | S | 1** | .033 | 0 | .967 | 5.419 |
| 4717 | S | 1** | .021 | 0 | .979 | 6.763 |
| 4749 | S | 1** | .041 | 0 | .959 | 4.812 |
| 4907 | S | 1** | .034 | 0 | .966 | 5.336 |
| 4977 | S | 1** | .017 | 0 | .983 | 7.508 |
| 4988 | S | 1** | .043 | 0 | .957 | 4.739 |
| 5055 | S | 1** | .021 | 0 | .979 | 6.763 |
| 5174 | S | 1** | .040 | 0 | .960 | 4.915 |
| 5175 | S | 1** | .034 | 0 | .966 | 5.336 |
| 5280 | S | 1** | .023 | 0 | .977 | 6.565 |
| 5324 | S | 1** | [illegible] | 0 | .983 | 7.508 |
| 5394 | S | 1** | [illegible] | 0 | .959 | 4.829 |
| 5433 | S | 1** | [illegible] | 0 | .957 | 4.739 |
| 5443 | S | 1** | .043 | 0 | .957 | 4.739 |
| 5507 | S | 1** | .034 | 0 | .966 | 5.336 |
| 5616 | S | 1** | .033 | 0 | .967 | 5.425 |
| 5681 | S | 1** | .041 | 0 | .959 | 4.812 |
| 5689 | S | 1** | .023 | 0 | .977 | 6.565 |
| 5786 | S | 1** | .041 | 0 | .959 | 4.808 |
| 5807 | S | 1** | .033 | 0 | .967 | 5.419 |
| 5929 | S | 1** | .041 | 0 | .959 | 4.808 |
| 5933 | S | 1** | .033 | 0 | .967 | 5.425 |
| 5971 | S | 1** | .041 | 0 | .959 | 4.829 |
| 6533 | S | 1** | .034 | 0 | .966 | 5.342 |
| 6706 | S | 1** | .016 | 0 | .984 | 7.863 |
| 6901 | S | 1** | .033 | 0 | .967 | 5.425 |
| 7124 | S | 1** | .040 | 0 | .960 | 4.915 |
| 7240 | S | 1** | .040 | 0 | .960 | 4.882 |
| 7271 | S | 1** | .040 | 0 | .960 | 4.882 |
| 7424 | S | 1** | .016 | 0 | .984 | 7.863 |
| 7520 | S | 1** | .041 | 0 | .959 | 4.812 |
| 7564 | S | 1** | .016 | 0 | .984 | 7.863 |
| 7656 | S | 1** | .033 | 0 | .967 | 5.384 |
| 7726 | S | 1** | .043 | 0 | .957 | 4.739 |

G-3

| | | | | | | |
|---|---|---|---|---|---|---|
| 7726 | S | 1** | .043 | 0 | .957 | 4.739 |
| 7789 | S | 1** | .034 | 0 | .966 | 5.342 |
| 7806 | S | 1** | .040 | 0 | .960 | 4.915 |
| 7834 | S | 1** | .017 | 0 | .983 | 7.508 |
| 7866 | S | 1** | .033 | 0 | .967 | 5.384 |
| 7877 | S | 1** | .041 | 0 | .959 | 4.829 |
| 8086 | S | 1** | .021 | 0 | .979 | 6.778 |
| 8258 | S | 1** | [illegible] | 0 | .984 | 7.811 |
| 8427 | S | 1** | [illegible] | 0 | .958 | 4.802 |
| 8448 | S | 1** | [illegible] | 0 | .981 | 7.202 |
| 8481 | S | 1** | .020 | 0 | .980 | 6.982 |
| 8483 | S | 1** | .041 | 0 | .959 | 4.845 |
| 8780 | S | 1** | .032 | 0 | .968 | 5.467 |
| 8892 | S | 1** | .015 | 0 | .985 | 7.994 |
| 8949 | S | 1** | .017 | 0 | .983 | 7.508 |
| 8994 | S | 1** | .017 | 0 | .983 | 7.508 |
| 9048 | S | 1** | .019 | 0 | .981 | 7.098 |
| 9175 | S | 1** | .017 | 0 | .983 | 7.508 |
| 9363 | S | 1** | .039 | 0 | .961 | 4.958 |
| 9383 | S | 1** | .032 | 0 | .968 | 5.467 |
| 9562 | S | 1** | .034 | 0 | .966 | 5.295 |
| 9781 | S | 1** | .021 | 0 | .979 | 6.876 |
| 10021 | S | 1** | .015 | 0 | .985 | 7.994 |

a. S = Selected, U = Unselected cases, and ** = Misclassified cases.
b. Cases with studentized residuals greater than 2.500 are listed.

**Casewise List[b]**

行 101 - 137 / 137

G-4

| Case | Selected Status[a] | Observed | Predicted | Predicted Group | Temporary Variable | |
|---|---|---|---|---|---|---|
| | | Macrosomia | | | Resid | ZResid |
| 10036 | S | 1** | .040 | 0 | .960 | 4.926 |
| 10064 | S | 1** | .031 | 0 | .969 | 5.546 |
| 10156 | S | 1** | .042 | 0 | .958 | 4.802 |
| 10188 | S | 1** | .019 | 0 | .981 | 7.098 |
| 10285 | S | 1** | .023 | 0 | .977 | 6.515 |
| 10328 | S | 1** | .041 | 0 | .959 | 4.855 |
| 10431 | S | 1** | .032 | 0 | .968 | 5.509 |
| 10549 | S | 1** | .016 | 0 | .984 | 7.872 |
| 10597 | S | 1** | .016 | 0 | .984 | 7.811 |
| 10769 | S | 1** | .020 | 0 | .980 | 6.982 |
| 10819 | S | 1** | .039 | 0 | .961 | 4.958 |
| 10872 | S | 1** | .014 | 0 | .986 | 8.364 |
| 10879 | S | 1** | .043 | 0 | .957 | 4.739 |
| 10949 | S | 1** | .021 | 0 | .979 | 6.876 |
| 10989 | S | 1** | .041 | 0 | .959 | 4.845 |
| 11086 | S | 1** | .031 | 0 | .969 | 5.546 |
| 11114 | S | 1** | .016 | 0 | .984 | 7.872 |
| 11317 | S | 1** | [illegible] | 0 | .959 | 4.855 |
| 11523 | S | 1** | [illegible] | 0 | .986 | 8.364 |
| 11738 | S | 1** | [illegible] | 0 | .977 | 6.515 |
| 11872 | S | 1** | [illegible] | 0 | .967 | 5.384 |
| 11966 | S | 1** | .044 | 0 | .956 | 4.671 |
| 12173 | S | 1** | .044 | 0 | .956 | 4.671 |
| 12177 | S | 1** | .040 | 0 | .960 | 4.926 |
| 12468 | S | 1** | .019 | 0 | .981 | 7.202 |
| 12668 | S | 1** | .034 | 0 | .966 | 5.342 |
| 12871 | S | 1** | .021 | 0 | .979 | 6.876 |
| 12972 | S | 1** | .034 | 0 | .966 | 5.295 |
| 13069 | S | 1** | .043 | 0 | .957 | 4.739 |
| 13114 | S | 1** | .032 | 0 | .968 | 5.509 |
| 13156 | S | 1** | .043 | 0 | .957 | 4.739 |
| 13252 | S | 1** | .034 | 0 | .966 | 5.342 |
| 13311 | S | 1** | .033 | 0 | .967 | 5.384 |
| 13713 | S | 1** | .032 | 0 | .968 | 5.467 |
| 13785 | S | 1** | .038 | 0 | .962 | 5.017 |
| 13974 | S | 1** | .032 | 0 | .968 | 5.467 |
| 14085 | S | 1** | .038 | 0 | .962 | 5.017 |

a. S = Selected, U = Unselected cases, and ** = Misclassified cases.
b. Cases with studentized residuals greater than 2.500 are listed.

H-1

**Casewise List**[b]

| Case | Selected Status[a] | Observed Macrosomia | Predicted | Predicted Group | Temporary Variable Resid | Temporary Variable ZResid |
|---|---|---|---|---|---|---|
| 202 | S | 1** | .037 | 0 | .963 | 5.076 |
| 337 | S | 1** | .019 | 0 | .981 | 7.236 |
| 345 | S | 1** | .041 | 0 | .959 | 4.807 |
| 359 | S | 1** | .042 | 0 | .958 | 4.804 |
| 454 | S | 1** | .029 | 0 | .971 | 5.757 |
| 624 | S | 1** | .041 | 0 | .959 | 4.807 |
| 795 | S | 1** | .032 | 0 | .968 | 5.527 |
| 917 | S | 1** | .021 | 0 | .979 | 6.823 |
| 934 | S | 1** | .024 | 0 | .976 | 6.323 |
| 1022 | S | 1** | .037 | 0 | .963 | 5.071 |
| 1070 | S | 1** | .040 | 0 | .960 | 4.897 |
| 1185 | S | 1** | .032 | 0 | .968 | 5.527 |
| 1412 | S | 1** | .040 | 0 | .960 | 4.897 |
| 1415 | S | 1** | .024 | 0 | .976 | 6.323 |
| 1497 | S | 1** | .029 | 0 | .971 | 5.757 |
| 1720 | S | 1** | [illegible] | 0 | .981 | 7.236 |
| 1799 | S | 1** | [illegible] | 0 | .981 | 7.096 |
| 1972 | S | 1** | [illegible] | 0 | .981 | 7.236 |
| 2155 | S | 1** | .021 | 0 | .979 | 6.823 |
| 2323 | S | 1** | .037 | 0 | .963 | 5.071 |
| 2375 | S | 1** | .021 | 0 | .979 | 6.823 |
| 2541 | S | 1** | .037 | 0 | .963 | 5.076 |
| 2697 | S | 1** | .019 | 0 | .981 | 7.096 |
| 2795 | S | 1** | .037 | 0 | .963 | 5.076 |
| 2824 | S | 1** | .040 | 0 | .960 | 4.897 |
| 2973 | S | 1** | .042 | 0 | .958 | 4.804 |
| 3111 | S | 1** | .037 | 0 | .963 | 5.071 |
| 3144 | S | 1** | .039 | 0 | .961 | 4.937 |
| 3157 | S | 1** | .033 | 0 | .967 | 5.431 |
| 3215 | S | 1** | .020 | 0 | .980 | 6.933 |
| 3261 | S | 1** | .033 | 0 | .967 | 5.431 |
| 3315 | S | 1** | .039 | 0 | .961 | 4.937 |
| 3494 | S | 1** | .037 | 0 | .963 | 5.111 |
| 3540 | S | 1** | .020 | 0 | .980 | 6.933 |
| 3585 | S | 1** | .020 | 0 | .980 | 6.933 |
| 3692 | S | 1** | .033 | 0 | .967 | 5.431 |
| 3812 | S | 1** | .037 | 0 | .963 | 5.111 |

H-2

| | | | | | | |
|---|---|---|---|---|---|---|
| 3812 | S | 1** | .037 | 0 | .963 | 5.111 |
| 3901 | S | 1** | .037 | 0 | .963 | 5.071 |
| 3921 | S | 1** | .037 | 0 | .963 | 5.111 |
| 4305 | S | 1** | .015 | 0 | .985 | 7.994 |
| 4358 | S | 1** | .015 | 0 | .985 | 7.994 |
| 4456 | S | 1** | .041 | 0 | .959 | 4.811 |
| 4528 | S | 1** | .021 | 0 | .979 | 6.787 |
| 4673 | S | 1** | .034 | 0 | .966 | 5.358 |
| 4690 | S | 1** | .033 | 0 | .967 | 5.420 |
| 4717 | S | 1** | .022 | 0 | .978 | 6.733 |
| 4749 | S | 1** | .041 | 0 | .959 | 4.811 |
| 4907 | S | 1** | .034 | 0 | .966 | 5.336 |
| 4977 | S | 1** | .017 | 0 | .983 | 7.497 |
| 4988 | S | 1** | .043 | 0 | .957 | 4.737 |
| 5055 | S | 1** | .022 | 0 | .978 | 6.733 |
| 5174 | S | 1** | .040 | 0 | .960 | 4.910 |
| 5175 | S | 1** | .034 | 0 | .966 | 5.336 |
| 5280 | S | 1** | .023 | 0 | .977 | 6.553 |
| 5324 | S | 1** | [illegible] | 0 | .983 | 7.497 |
| 5394 | S | 1** | [illegible] | 0 | .958 | 4.796 |
| 5433 | S | 1** | [illegible] | 0 | .957 | 4.737 |
| 5443 | S | 1** | .043 | 0 | .957 | 4.737 |
| 5507 | S | 1** | .034 | 0 | .966 | 5.336 |
| 5616 | S | 1** | .033 | 0 | .967 | 5.441 |
| 5681 | S | 1** | .041 | 0 | .959 | 4.811 |
| 5689 | S | 1** | .023 | 0 | .977 | 6.553 |
| 5786 | S | 1** | .041 | 0 | .959 | 4.816 |
| 5807 | S | 1** | .033 | 0 | .967 | 5.420 |
| 5929 | S | 1** | .041 | 0 | .959 | 4.816 |
| 5933 | S | 1** | .033 | 0 | .967 | 5.441 |
| 5971 | S | 1** | .042 | 0 | .958 | 4.796 |
| 6533 | S | 1** | .034 | 0 | .966 | 5.358 |
| 6706 | S | 1** | .016 | 0 | .984 | 7.854 |
| 6901 | S | 1** | .033 | 0 | .967 | 5.441 |
| 7124 | S | 1** | .040 | 0 | .960 | 4.910 |
| 7240 | S | 1** | .040 | 0 | .960 | 4.891 |
| 7271 | S | 1** | .040 | 0 | .960 | 4.891 |
| 7424 | S | 1** | .016 | 0 | .984 | 7.854 |
| 7520 | S | 1** | .041 | 0 | .959 | 4.811 |
| 7564 | S | 1** | .016 | 0 | .984 | 7.854 |
| 7656 | S | 1** | .033 | 0 | .967 | 5.400 |

H-3

| | | | | | | |
|---|---|---|---|---|---|---|
| 7656 | S | 1** | .033 | 0 | .967 | 5.400 |
| 7726 | S | 1** | .043 | 0 | .957 | 4.737 |
| 7789 | S | 1** | .034 | 0 | .966 | 5.358 |
| 7806 | S | 1** | .040 | 0 | .960 | 4.910 |
| 7834 | S | 1** | .017 | 0 | .983 | 7.497 |
| 7866 | S | 1** | .033 | 0 | .967 | 5.400 |
| 7877 | S | 1** | .042 | 0 | .958 | 4.796 |
| 8086 | S | 1** | .021 | 0 | .979 | 6.787 |
| 8258 | S | 1** | .016 | 0 | .984 | 7.824 |
| 8427 | S | 1** | .042 | 0 | .958 | 4.797 |
| 8448 | S | 1** | .019 | 0 | .981 | 7.227 |
| 8481 | S | 1** | [illegible] | 0 | .980 | 6.972 |
| 8483 | S | 1** | [illegible] | 0 | .959 | 4.853 |
| 8780 | S | 1** | [illegible] | 0 | .968 | 5.484 |
| 8892 | S | 1** | .015 | 0 | .985 | 8.008 |
| 8949 | S | 1** | .017 | 0 | .983 | 7.497 |
| 8994 | S | 1** | .017 | 0 | .983 | 7.497 |
| 9048 | S | 1** | .019 | 0 | .981 | 7.109 |
| 9175 | S | 1** | .017 | 0 | .983 | 7.497 |
| 9363 | S | 1** | .039 | 0 | .961 | 4.968 |
| 9383 | S | 1** | .032 | 0 | .968 | 5.484 |
| 9562 | S | 1** | .034 | 0 | .966 | 5.295 |
| 9781 | S | 1** | .021 | 0 | .979 | 6.865 |
| 10021 | S | 1** | .015 | 0 | .985 | 8.008 |

a. S = Selected, U = Unselected cases, and ** = Misclassified cases.
b. Cases with studentized residuals greater than 2.500 are listed.

行 1 - 100 / 143

H-4

行 101 - 143 / 143

| Case | Selected Status[a] | Observed Macrosomia | Predicted | Predicted Group | Temporary Variable Resid | Temporary Variable ZResid |
|---|---|---|---|---|---|---|
| 10036 | S | 1** | .039 | 0 | .961 | 4.949 |
| 10064 | S | 1** | .031 | 0 | .969 | 5.547 |
| 10156 | S | 1** | .042 | 0 | .958 | 4.797 |
| 10188 | S | 1** | .019 | 0 | .981 | 7.109 |
| 10285 | S | 1** | .023 | 0 | .977 | 6.503 |
| 10328 | S | 1** | .040 | 0 | .960 | 4.868 |
| 10431 | S | 1** | .032 | 0 | .968 | 5.526 |
| 10549 | S | 1** | .016 | 0 | .984 | 7.885 |
| 10597 | S | 1** | .016 | 0 | .984 | 7.824 |
| 10769 | S | 1** | .020 | 0 | .980 | 6.972 |
| 10819 | S | 1** | .039 | 0 | .961 | 4.968 |
| 10872 | S | 1** | .014 | 0 | .986 | 8.397 |
| 10879 | S | 1** | .043 | 0 | .957 | 4.737 |
| 10949 | S | 1** | .021 | 0 | .979 | 6.865 |
| 10989 | S | 1** | .041 | 0 | .959 | 4.853 |
| 11086 | S | 1** | .031 | 0 | .969 | 5.547 |
| 11114 | S | 1** | .016 | 0 | .984 | 7.885 |
| 11317 | S | 1** | .040 | 0 | .960 | 4.868 |
| 11523 | S | 1** | .014 | 0 | .986 | 8.397 |
| 11738 | S | 1** | .023 | 0 | .977 | 6.503 |
| 11872 | S | 1** | .033 | 0 | .967 | 5.400 |
| 11966 | S | 1** | .044 | 0 | .956 | 4.683 |
| 12173 | S | 1** | .044 | 0 | .956 | 4.683 |
| 12177 | S | 1** | .039 | 0 | .961 | 4.949 |
| 12468 | S | 1** | .019 | 0 | .981 | 7.227 |
| 12668 | S | 1** | .034 | 0 | .966 | 5.358 |
| 12871 | S | 1** | .021 | 0 | .979 | 6.865 |
| 12972 | S | 1** | .034 | 0 | .966 | 5.295 |
| 13069 | S | 1** | .043 | 0 | .957 | 4.737 |
| 13114 | S | 1** | .032 | 0 | .968 | 5.526 |
| 13156 | S | 1** | .043 | 0 | .957 | 4.737 |
| 13252 | S | 1** | .034 | 0 | .966 | 5.358 |
| 13311 | S | 1** | .033 | 0 | .967 | 5.400 |
| 13713 | S | 1** | .032 | 0 | .968 | 5.484 |
| 13785 | S | 1** | .038 | 0 | .962 | 5.016 |
| 13974 | S | 1** | .032 | 0 | .968 | 5.484 |
| 14085 | S | 1** | .038 | 0 | .962 | 5.016 |
| 14541 | S | 1** | .042 | 0 | .958 | 4.769 |
| 14560 | S | 1** | .043 | 0 | .957 | 4.732 |
| 14614 | S | 1** | .042 | 0 | .958 | 4.769 |

H-5

| | | | | | | |
|---|---|---|---|---|---|---|
| 14614 | S | 1** | [illegible] | 0 | .958 | 4.769 |
| 14712 | S | 1** | [illegible] | 0 | .957 | 4.732 |
| 14729 | S | 1** | [illegible] | 0 | .957 | 4.695 |
| 14791 | S | 1** | .043 | 0 | .957 | 4.695 |

a. S = Selected, U = Unselected cases, and ** = Misclassified cases.
b. Cases with studentized residuals greater than 2.500 are listed.

G-1, G-2, G-3, G-4: Model 1 in Table 4; H-1, H-2, H-3, H-4, H-5: Model 2 in Table 4.

The most extreme values in the data can be examined by visualizing casewise listing of residuals. Here we labelled the top 2.5 largest values in the casewise list, and solved by removing the concerned records.

All data were analyzed by SPSS statistical software v19.0.
